# ASSESSMENT OF BODY FAT IN OBESE PATIENTS PREOPERATIVELY FOR BARIATRIC SURGERY

**DOI:** 10.1590/0102-6720201600S10015

**Published:** 2016

**Authors:** Mônica FERNANDEZ, Rosana Farah TOIMIL, Zied RASSLAN, Elias Jirjoss ILIAS, Ana Lúcia Torloni GRADINAR, Carlos Alberto MALHEIROS

**Affiliations:** Faculty of Medical Sciences, Santa Casa de São Paulo, São Paulo, SP, Brazil.

**Keywords:** Anthropometry, Bariatric surgery, Body composition

## Abstract

**Background:**

The study of body composition in patient candidates for bariatric surgery is directly related to the increase and distribution of body fat in the development of cardiovascular disease.

**Aim:**

To correlate anthropometric indicators and bioelectrical impedance in the assessment of body fat in female candidates for bariatric surgery.

**Methods:**

Cross-sectional, observational study of 88 women. The weight, height, body mass index and waist circumference data were evaluated in the anthropometric analysis. The body fat was determinate by bioelectrical impedance conducted according to the manufacturer´s recommended technique with a specific severe obesity formula. The patients were divided into two subgroups according to the average waist circumference and body mass index for better analysis of the results.

**Results:**

The group had a mean age of 39.7 years (±7.2), average weight of 125.6 kg (±16.2), mean body mass index of 48.7 kg/m^2^ (±6.4) and the mean waist circumference 137.6 cm (±12.4). Negative and significant relationship between BMI values waist circumference and resistance obtained by bioelectrical impedance ​​were found. By analyzing the two groups the mean BMI and waist circumference, a significant relationship was observed, ie, the higher the degree of obesity less resistance was obtained by bioelectrical impedance. The higher is the obesity the lower is value found for resistance.

**Conclusion:**

The increase of anthropometric indicators (BMI and waist circumference) determined reduction in resistance and reactance obtained by bioelectrical impedance analysis in obese women candidates to bariatric surgery.

## RESUMO

**Racional:**

O estudo da composição corporal em pacientes candidatas à cirurgia bariátrica tem relação direta com o aumento e distribuição da gordura corporal e no desenvolvimento de doenças cardiovasculares.

**Objetivo:**

Estudar a correlação entre indicadores antropométricos e da bioimpedância elétrica na avaliação da gordura corporal em mulheres candidatas à cirurgia bariátrica.

**Métodos:**

Estudo transversal, observacional com 88 pacientes. Na análise antropométrica foram avaliados os dados de peso, altura, IMC e circunferência da cintura. Para bioimpedância elétrica utilizou-se a técnica recomendada pelo fabricante do aparelho e fórmula específica para obeso grave na determinação da gordura corporal. Para melhor análise dos resultados as pacientes foram divididas em dois subgrupos de acordo com a média de circunferência da cintura e IMC.

**Resultados:**

O grupo apresentou média de idade 39,7 anos (±7,2), média de peso 125,6 kg (±16,2), média de IMC 48,7 kg/m^2^ (±6,4) e média de circunferência da cintura 137,6 cm (±12,4). Foram encontradas relações negativas e significativas entre os valores de IMC, circunferência da cintura e a resistência obtida através da bioimpedância elétrica. Ao analisar os dois subgrupos de valores médios de IMC e circunferência da cintura, observou-se relação significativa, ou seja, quanto maior o grau de obesidade menor é a resistência obtida através da bioimpedância elétrica.

**Conclusão:**

O aumento dos indicadores antropométricos (IMC e circunferência da cintura) determinaram redução nas variáveis resistência e reatância obtidas pela bioimpedância elétrica em mulheres candidatas à cirurgia bariátrica.

## INTRODUCTION

Obesity has a multifactorial etiology including genetic predisposition, environmental and behavioral factors^19^. Its increase is of great importance as a public health problem in modern society^14^. In studies on the quality of life of patients eligible for bariatric surgery, Costa et al. reported that obesity interferes in physical, emotional, psychological and social aspects^5^. Body composition estimation is in constant concern in relation to the increase in body fat, and its distribution, in relationship to cardiovascular diseases^8^ ^,^ ^14^. The assessment of body composition can be performed with the use of anthropometric indicators, such as weight, height and waist circumference, and by technical devices as bioelectrical impedance^8^. 

Anthropometry is inexpensive method, easy to apply and equations exist in order to predict the body composition using height, weight and waist circumference^7^. According to authors, waist circumference has higher correlation with visceral adiposity when compared to MRI and CT^11^. 

To stratify the body composition, as well as the risk for development of diseases, World Health Organization, (WHO) has proposed the classification based on BMI that is represented by the ratio of weight in kilograms and height in square meters^15^; in clinical practice it is considered simple, fast and reproducible^21^.

The use of bioelectrical impedance as instrumental method for assessing body composition - based on the measurement of total body resistance to an electric current of low amplitude (800uA) and high frequency (50kHz) - allows measurement of the resistance and the reactance^9^ ^,^ ^11^. Resistance is pure restriction to the flow of an electrical current through the body, related to the extra and intracellular fluids. The resistance is inversely proportional to the amount of body water. The reactance means the opposition of the electrical flow caused by the capacitance produced by tissues and cell membranes, reflects the ability of the membrane to act as capacitors^9^ ^,^ ^11^. The relationship between reactance and resistance reflects different electrical properties of tissues; the results may be affected in various ways by disease, nutritional status and degree of hydration. From the values ​​of resistance and reactance, are used predictive equations to define the percentage of body fat. In patients with obesity, studies recommend the use of the equation of Horie-Waitzberg and Barbosa-Silva (HW&BS)^4^ ^,^ ^9^ ^,^ ^11^.

The scientific literature on body composition in patients with severe obesity is very rare^11^. The evaluation of the fat in these patients may contribute to better understanding of risks for cardiovascular diseases^13^ ^,^ ^16^.

This study aimed to evaluate body fat through anthropometric indicators and bioelectrical impedance of women candidates for bariatric surgery. 

## METHODS

Were enrolled 88 women in preoperative evaluation at High Complexity Assistance to Obesity Specialties Clinic of Santa Casa de São Paulo, São Paulo, SP, Brazil. This study was approved by the Research Ethics Committee of the institution under number CAAE: 25071913.1.0000.5479. After signing the free and informed consent, patients underwent anthropometric measurement and bioelectrical impedance.

All evaluations were made based on technical proposals by the Ministry of Health^3^. According to recommendations of Van Der Kooy and Seidell, the measure of waist circumference for patients with severe obesity should be at the umbilicus^20^, and considered as cutoff point for cardiovascular risk being it equal to or greater than 80 cm in women^3^.

For the estimation of body composition was used a portable bioelectrical impedance device model Quantum II, RJL Systems(r), with frequency of 50 kHz, following the method recommended by the manufacturer. From the data provided by bioelectrical impedance device were obtained body fat values according to the equation HW & BS^1^ ^,^ ^9^ ^,^ ^10^. 

Was also calculated the percentage of body fat values from equations already programmed by the manufacturer of the instrument itself. As normal range for body fat percentage, was considered value of 20-25%^2^ ^,^ ^6^ ^,^ ^9^ ^,^ ^17^.

Patients eligible to participate in the study had to attend the following inclusion criteria: age lower than 20 years and BMI ≥40 kg/m². Were excluded patients with acute or chronic disease, in pre- or menopause stage, and in use of medications to promote weight loss.

### Statistical analysis

It has been used the Pearson correlation method for the correlations of the variables, and the Fisher test for analysis of variance. For better statistical analysis of the results obtained with the bioelectrical impedance for the values of resistance and reactance, it was decided to stratify patients into two subgroups, according to the average waist circumference (137cm) and body mass index (49 kg/m²). For this analysis was used the Mann-Whitney test.

## RESULTS

48% of the patients had a mean age of 39.7 years, and most unmarried; 42% were between 12-16 years of schooling; 61% had no history of previous pregnancy; and 73% worked outside home. The mean BMI was 48.7 kg/m² and the waist circumference was 137.6 cm (Table 1).

**TABLE 1:** Description of anthropometric variables of women candidates for bariatric surgery (n = 88)

Variables	Mean±standard deviation
Current weight (kg)	125,6±16,2
Height (cm)	160,6±6,2
BMI (Kg/m²)	48,7±6,4
Waist circumference (cm)	137,6±12,4

The waist circumference values were greater than 80 cm in 100% of patients and 75% had values greater than 131 cm. As for the percentage of BMI found in the study group, it was observed that 34 (38.6%) had values between 40-45 kg/m², 43 (49%) between 46-55 kg/m², and 11(12.5%) >56 kg/m².

The average percentage of body fat found by bioelectrical impedance and the equation of HW&BS, showed no significant differences; percentage difference between the two formulas was found only in 0.9% (Table 2).

**TABLE 2:** Mean percentage of body fat found by the formula of the bioelectrical impedance equipment and the HW & BS equation

	Mean	±DP
Formula BIA	49,5%	±4,67
Formula HW&BS	48,6%	± 5,80

There was a negative and significant correlation between BMI/waist circumference and the resistance/reactance values obtained by bioelectrical impedance analysis (Table 3).

**TABLE 3:** Distribution values of the coefficient of correlation

Variables	Correlation	Resistance	Reactance
Body mass index	Correlation coefficient p value	-0,441 0,000	-3,396 0,000
Waist circumference	Correlation coefficient p value	-0,464 0,000	-0,352 0,001

significant p<0.05

Was chosen to stratify the study patients into two subgroups according to the average waist circumference and BMI (Table 4). By analyzing the two groups the mean BMI and waist circumference, a significant relationship was observed, ie, the higher the degree of obesity lower was the resistance obtained by bioelectrical impedance.

**TABLE 4:** Correlation between the two subgroups of waist circumference and BMI and the variables obtained in bioelectrical impedance

Variables	Waist circumference	Mean ±DP	p	BMI	Mean ±DP	p
Resistance	<137	422±46,9	0,001	< 49	420±49,6	0,001
	≥137	385±50,8		≥ 49	381±47,5	
Reatance	< 137	50,9±6,27	0,002	< 49	50,9±5,9	<0,001
	≥137	46,4±6,08		≥ 49	45,6±6,18	

significant p < 0.05

## DISCUSSION

The results on the demographic profile in this study were similar to those observed in other studies^12^ ^,^ ^18^. Here, 100% of patients had a circumference value higher than 80 cm. It was already said that body composition distribution of fat is as important as the severity of obesity. Patients with central adiposity have higher risk of developing cardiovascular complications from obesidade^19^. 

Researchers refer the importance of using specific equation for each study group to determine the percentage of body fat by bioelectrical impedance^2^ ^,^ ^6^ ^,^ ^9^ ^,^ ^17^. In this study was used the equation HW&BS and compared to the results obtained with the equation already set by the manufacturer of the device. There was no significant difference. Perhaps this result is related to the distribution of body fat and intra and extracellular fluids in various degrees of obesity, which is a limitation to the use of HW & BS equation^9^.

Limiting factor for assess body fat in this group by bioelectrical impedance, was the distribution between the cellular liquids in various degrees of obesity; studies show that chronic inflammation is an important aspect linked to obesity and that the balance of body fluids are strongly affected in the presence of inflammation^9^. The scientific knowledge of the distribution of body components, such as lean mass, body fat percentage and intra and extracellular liquids, are very poor, a fact that made it difficult to discuss these results^2^. Knowledge on body fat distribution, as well as on risk factors in the severe obesity, is important to reduce pre- and postoperative risks in candidates for bariatric surgery.

## CONCLUSION

The increase of anthropometric indicators (BMI and waist circumference) determined reduction in resistance and reactance obtained by bioelectrical impedance analysis in obese women candidates to bariatric surgery.
